# Degradation of Oxo-Biodegradable Plastic by *Pleurotus ostreatus*

**DOI:** 10.1371/journal.pone.0069386

**Published:** 2013-08-15

**Authors:** José Maria Rodrigues da Luz, Sirlaine Albino Paes, Mateus Dias Nunes, Marliane de Cássia Soares da Silva, Maria Catarina Megumi Kasuya

**Affiliations:** Departamento de Microbiologia, Universidade Federal de Viçosa, Viçosa, Minas Gerais, Brazil; French National Centre for Scientific Research, France

## Abstract

Growing concerns regarding the impact of the accumulation of plastic waste over several decades on the environmental have led to the development of biodegradable plastic. These plastics can be degraded by microorganisms and absorbed by the environment and are therefore gaining public support as a possible alternative to petroleum-derived plastics. Among the developed biodegradable plastics, oxo-biodegradable polymers have been used to produce plastic bags. Exposure of this waste plastic to ultraviolet light (UV) or heat can lead to breakage of the polymer chains in the plastic, and the resulting compounds are easily degraded by microorganisms. However, few studies have characterized the microbial degradation of oxo-biodegradable plastics. In this study, we tested the capability of *Pleurotus ostreatus* to degrade oxo-biodegradable (D_2_W) plastic without prior physical treatment, such as exposure to UV or thermal heating. After 45 d of incubation in substrate-containing plastic bags, the oxo-biodegradable plastic, which is commonly used in supermarkets, developed cracks and small holes in the plastic surface as a result of the formation of hydroxyl groups and carbon-oxygen bonds. These alterations may be due to laccase activity. Furthermore, we observed the degradation of the dye found in these bags as well as mushroom formation. Thus, *P. ostreatus* degrades oxo-biodegradable plastics and produces mushrooms using this plastic as substrate.

## Introduction

Pollutants composed of plastic polymers have been removed from the environment using three approaches. The first approach is to store the waste in landfills. However, due to rapid population growth and the limited number of landfills, this approach only transfers the problem to future generations [1], [2]. Plastic waste represents 20–30% of the total volume of solid waste contained in landfills because in addition to the large amount of waste generated, plastic waste is recalcitrant and remains deposited in these landfills for long periods of time [3]. The second approach can be subdivided into two distinct parts: incineration and recycling [2], [4]. The incineration of plastic waste often results in a significant release of carbon dioxide and other gases. The recycling process includes removing the plastic residue, separating plastic into categories according to type, and washing, drying, grinding and reprocessing the plastic waste [2]. Thus, recycling is an expensive process, and the quality of the recycled plastic is lower than that of the primary material [1]–[3]. The final approach is the development of biodegradable polymers.

Biodegradable plastics can be divided into three groups according to their origin: (a) bacterial polymers that can be formed by a bacterial biofilm or by microbial fermentation, (b) polymers that are derived from plants and (c) chemically synthesized polymers [3]. Biodegradable polymers derived from renewable sources, such as plants or microorganisms, are ecologically maintainable because they are not accumulated in the environment for long periods of time and they are degraded or mineralized by microorganisms. However, these polymers contain some physicochemical properties that restrict their use [5].

Biodegradable plastics that are synthesized by chemical modifications may be divided into two groups: (a) those obtained by the degradation of chemical structures by the direct action of enzymes, such as amylase and cellulase, and (b) those that are made degradable by the action of one or various physic-chemical processes, for example, hydrolysis, photolysis or pyrolysis [4], [5].

The polymers of the second subgroup are designated as oxo-biodegradable polymers, or D_2_W, due to the presence of the pro-oxidant or pro-degrading additives [1], [5]. The pro-oxidant additive is incorporated into the polymer chain and represents approximately 1–5% of the polymer molecular weight [5]. These pro-oxidants are based on combinations of metal ions of similar stability and oxidation number, for example, Co^+2^/Co^+3^, Mn^+2^/Mn^+3^, Fe^+2^/Fe^+3^ and TiO_2_ [4], [5]. The pro-oxidant that is embedded in the polymer chain accelerates the photo and thermal oxidation [4]. Thus, when these residues are exposed to UV or high temperatures, they are degraded by the formation of free radicals that react with atmospheric oxygen, leading to polymer chain scission and the production of low molecular weight compounds, such as carboxylic acids, alcohols and ketones [6]. These compounds are then assimilated by microorganisms [4].

In contrast to most synthetic polymers that are derived from petroleum, biodegradable polymers, when discarded in the environment, may initially be cleaved from the polymer chain by non-enzymatic processes, such as photolysis and chemical hydrolysis, and subsequently degraded by enzymes produced by algae, bacteria or fungi [7]. These biodegradable polymers can be converted to carbon dioxide, methane, water, biomass, humus and other substances [7]. Therefore, the aim of this study was to evaluate the capacity of *P. ostreatus* to degrade oxo-biodegradable plastic and to form mushrooms in this waste.

## Materials and Methods

### Microorganisms and cultivation conditions

The isolate *Pleurotus ostreatus* PLO6 (Laboratory of Mycorrhizal Associations/DMB/BIOAGRO/UFV) was grown in a Petri dish containing potato dextrose agar (PDA) at pH 5.8 and incubated at 25°C for 20 d. This isolate was obtained from a basidiocarp of commercial mushrooms that was produced in Moji das Cruzes, São Paulo, Brazil. In different lignocellulosic residues, this isolate forms the typical oyster mushroom of this genus [8], [9].

The molecular identification was performed with a total DNA extraction of 250 mg of fungal mycelium using a Spin Kit® Plant Mini Invisorb (Invetek, Berlin, Germany), according to the manufacturer's instructions. The DNA integrity was evaluated on a 0.8% (w/v) agarose gel, which was visualized and photographed using UV and the Molecular Imaging system (Loccus Biotecnologia L-Pix Chemi). The DNA was then subjected to PCR amplification, in which the reaction was prepared with 20.0 ng of total DNA, 0.2 µmol L^−1^ of each primer, 200 µM of dNTP, 2 mmol L^−1^ of MgCl_2_, 0.5 mg mL^−1^ of bovine serum albumin and 1.25 U Go Taq® DNA polymerase (Promega, Madison, USA). The primers ITS1 [10] and ITS4 [11] were used for the amplification of the fragments of rDNA 18S. The negative control was performed using 1 µL of sterile deionized water to replace the total DNA.

The PCR reaction was performed in a PCR Express thermocycler (Mastercycler epgradient, Eppendorf). The following thermocycling program was used: 95°C for 2 min; 95°C for 1 min (39 cycles); and 72°C for 1 min; followed by 72°C for 7 min. The success of the PCR amplification was tested on a 1.5% agarose gel that was stained in a solution of ethidium bromide (0.5 µg mL^−1^). The PCR products were visualized and photographed using UV and the Molecular Imaging system. The amplicon was sequenced by Macrogen Pathfinder Inc. Genomics Research (Seoul, Korea) using the Applied Biosystems 3730XL sequencers. To obtain a putative identification, this result was compared with sequences present in the Genbank NCBI using the BLASTn program at the NCBI homepage (http://www.ncbi.nlm.nih.gov/).

### Cultivation conditions

Plastic bags that are commonly used in supermarkets, with the description D_2_W or oxo-biodegradable, were cut into fragments (5 cm×1 cm), and 10 g of this material was placed in a glass flask (100 mL) with 0.1 g of a commercially available paper towel. Five milliliters of the mineral medium [4], [12] that was supplemented with sterile thiamine-HCl was added. Four discs of agar (6–8 mm) containing the mycelium of *P. ostreatus* were inoculated. These substrates were then incubated at 25°C for 45 d for fungal growth.

Flasks containing plastic strips with no addition of the paper towel were used as the control treatment. However, moisture loss occurred rapidly, and fungal growth was not observed. Therefore, this treatment was discarded, and the analyses described in this study were made using the treatment with the paper addition.

### Fungal biomass determination

The mycelial growth and visual changes on the plastic strips, including discoloration, were documented by digital photography.

The fungal biomass was determined by the ergosterol content [13]. To extract this compound, 3 g of the samples that were colonized by fungus was triturated for 10 min using a porcelain mortar. These samples were subsequently transferred to 50 mL centrifuge tubes containing 0.3 g of polyvinylpyrrolidone (Sigma) and 15 mL of ethanol 95% (Merck, Darmstadt, Germany). The samples were homogenized and centrifuged at 4000× *g* for 20 min at 4°C. The supernatants were filtered through a paper filter (Whatman GF/D, degree 2.5 cm). From these supernatants, 20 µL was used to determine the ergosterol content by high performance liquid chromatography (HPLC, Shimadzu – LC 10 A, C18 reverse phase and UV detection at 280 nm). The samples were eluted with 1.0 mL min^−1^ of methanol (Sigma) and ergosta-5,7,22-trien-3β-ol (Sigma) with concentrations ranging from 3 to 160 mg L^−1^ to construct a standard curve.

### Analysis of the biodegradation of the oxo-biodegradable polymers

Physical changes, such as the formation of holes and cracks, and the fungal colonization on the surface of the plastic waste were analyzed by scanning electron microscopy (Leo, 1430VP) with a magnification of 5000 [5], [12], [14].

The alteration of the mechanical properties of the plastic waste was analyzed using the universal testing equipment (Instron model 3367). The identification of the pro-oxidant was made using SEM coupled with X-ray diffraction.

Chemical changes, such as the disappearance or formation of new functional groups and bond scission, were analyzed by Fourier transform infrared spectroscopy (FTIR) [4], [15], [16]. The plastic strip fragment was placed in an appropriate support for solid sampling in the spectrophotometer (Thermo Nicolet).

### Enzymatic assays

To evaluate the lignocellulolytic enzyme production by *P. ostreatus* after 45 d of incubation in the substrate (oxo-biodegradable plastic strips and a paper towel), 3 g of each sample was placed in a 125 mL Erlenmeyer flask containing 15 mL of sodium citrate buffer (50 mM, pH 4.8). The flask was placed on a shaker for 30 min at 150 rpm, and the extract was filtered with Millipore membranes [17]. The enzyme assays described below were performed in triplicate.

Laccase and manganese peroxidase (MnP) activities were measured using 2,2′-azino-bis-3-etilbenzotiazol-6-sulfonic acid and phenol red solution as substrates [17], [18]. Xylanase and cellulase activities were calculated by measuring the levels of the reducing sugars that were produced during the enzymatic reactions [18]–[20]. One unit of enzyme activity was defined as the amount of enzyme required to catalyze the production of one µmol of colored product or reducing sugars per mL per min.

The level of reducing sugars was determined by the DNS method (99.5% dinitrosalicylic acid, 0.4% phenol and 0.14% sodium metabisulfite), and the standard curve was made using glucose or xylose (Merck, Darmstadt, Germany) with the concentrations of the standard ranging from 0.5 to 1.5 g L^−1^.

The content of the soluble protein was determined using the Bradford method [21]; this result was used to calculate the specific activity of the enzyme.

### Mushroom formation

To induce the formation of basidiocarps, the flasks containing the plastic waste, that were colonized by *P. ostreatus* mycelium, were transferred on the 40^th^ day of incubation to a chamber at 10±2°C. After 48 h, the flasks were held in another chamber at 18°C and a relative humidity of 80% [9].

## Results and Discussion

The nucleotide sequence obtained by the amplicon sequencing of the rDNA 18S of *P. ostreatus* PLO6 was deposited in GenBank (KC782771). The alignment of this sequence with other sequences found in the database showed a 92% similarity with the sequence EF514247.1, described as *Pleurotus ostreatus* strain CGMCC 5.37 [22]. Thus, the sequencing confirmed the previous morphologic classification of the isolate PLO6 as the species *P. ostreatus*. This classification was made based in the basidiocarps (mushrooms) of this fungus [8], [9].

The spectra obtained by FTIR were compared with the spectra contained in the library of the spectrophotometer. The plastic polymers that were used as the substrate to grow *P. ostreatus* were identified as low density polyethylene (Table 1, Figure S1). This technique was also used by Koutny et al. [4] and Sudhakar et al. [16] to identify plastic polymers. Mechanical properties, such as the maximum load of break, the tensile extension at break and the elastic modulus (Table 1), that were similar to those properties obtained by Coutinho et al. [23] confirmed the identification of the plastic polymers as low density polyethylene. Furthermore, the presence of titanium in the plastic polymers showed that these polymers are oxo-biodegradable (Table 1, Figure S2).

**Table 1:** Mechanical properties and identification of the polymers used to *P. ostreatus* PLO6 growth.

Polymer	Before of the incubation	After 45 days of incubation
Low density polyethylene*	***	see figure 3
Pro-oxidant**	Titanium oxide	nd
Mechanical properties		
Maximum load of break (N)	4.362±0.444	2.260±0.751
Energy at break (J)	0.069±0.023	0.013±0.004
Tensile extension at break (cm)	0.532±0.130	0.300±0.121
Load at tensile strength (N)	2.820±0.365	1.850±0.725
Elastic modulus (MPa)	33.341±3.341	17.417±5.777

* determined by Fourier transform infrared spectroscopy (see figure S1).

** determined by scanning electron microscopy coupled diffraction in ray X (see figure S2).

*** see figure S1 and figure S2.

nd – not determined.

**Figure 1:**
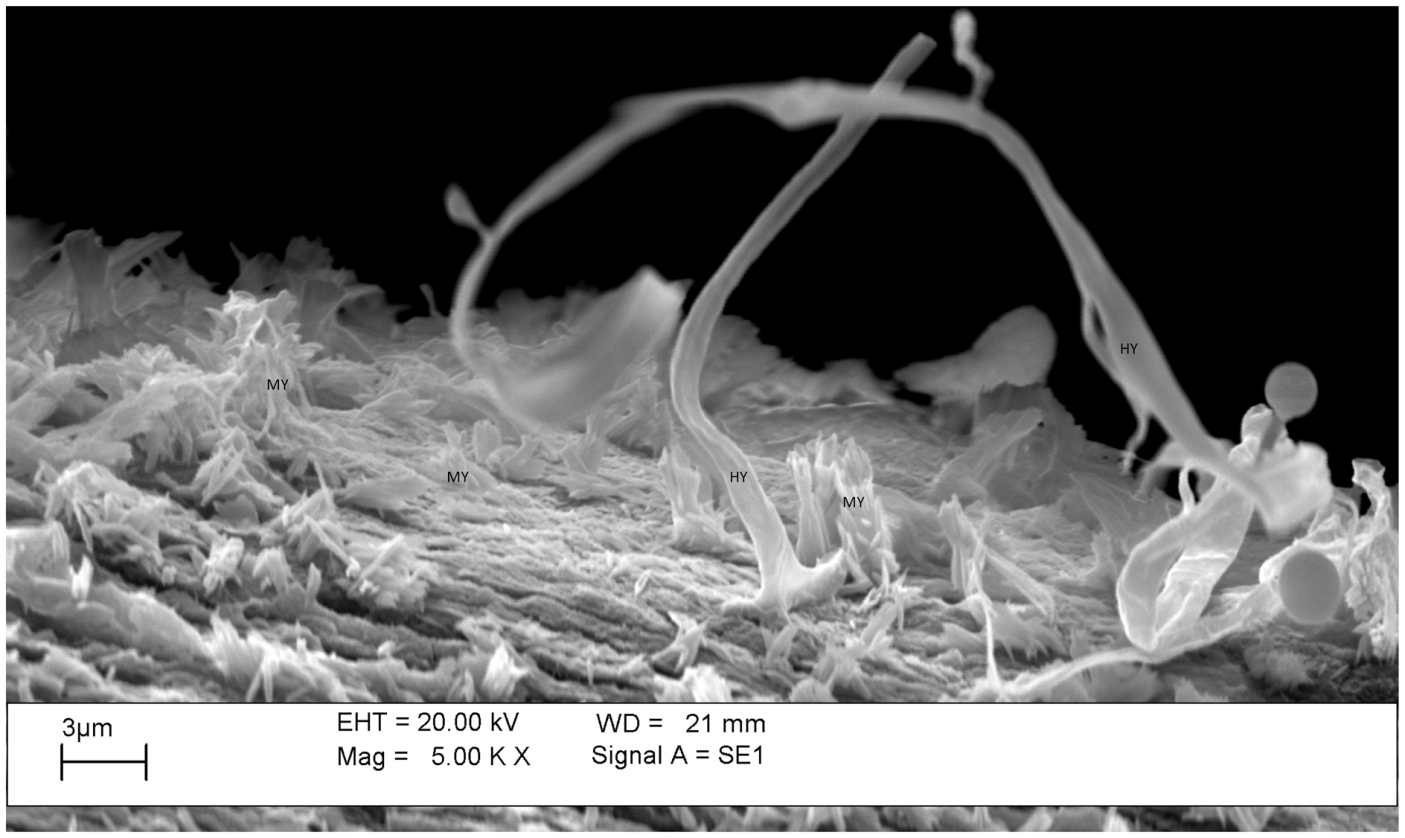
Scanning electron microscopy of *Pleurotus ostreatus* PLO6 growing on the surface of oxo-biodegradable plastic strip, after 45 days of incubation at 25°C. MY – Mycelium, HY – Hyphal.

In this work, we observed the mycelial growth on the surface of the plastic waste (Figure 1), which confirmed fungal biomass production by *P. ostreatus* on the oxo-biodegradable plastic (Table 2) without any prior physical treatment, such as exposure to UV or thermal heating. After 45 d of incubation, fungal colonization was observed on the surface of the oxo-biodegradable waste by scanning electron microscopy (Figure 1). Other studies have also shown microbial colonization [16], [24]; biofilm formation for *Rhodococcus rhodochrous*, *Cladosporium cladosporoides* and *Norcardia asteroids* [12]; and the growth of *Aspergillus flavus*, *Penicillium simplicissium* and *Phanerochaete chrysosporium* [4], [5], [15] in oxo-biodegradable polyethylene; however, each of these processes occurred after the sample was subjected to heat treatment or UV irradiation. Regardless of the need to apply physical treatment, these data show the capacity of microorganisms to use a synthetic polymer as a source of carbon and energy, thereby presenting an alternative for the degradation of these new plastic polymers with the biodegradation information.

**Table 2:** Specific activity of the ligninocellulolytic enzymes and fungal biomass of *Pleurotus ostreatus* PLO6, grown for 45 days in oxo-biodegradable.

Enzymes	Specific activity (U mg^−1^ dry mass)
Laccase	0.1855±0.005
Manganese peroxidase	nd
Cellulase	1.1294±0.231
Xylanase	0.5117±0,126
Biomass	µg mg^−1^ dry mass
Ergosterol	53.6327±5,4512

nd – not determined.

Similar to the observation of the polyethylene degradation by *P. simplicissimum* YK [25], we observed that *P. ostreatus* could use this polymer as a carbon source for biomass and metabolite production (Table 2). The ergosterol content produced by *P. ostreatus* confirms in this observation (Table 2). Ergosterol content can be used to monitor fungal biomass because this compound is labile and, therefore, is quickly degraded after hyphal death [17], [26]–[28]. Furthermore, the membrane lipid ergosterol is found almost exclusively in fungi [27], [28]. Barajas-Aceves et al. [26] observed that ergosterol content produced by white-rot fungi is independent of the presence or absence of different pollutants of the soil (e.g., pentachlorophenol, N,N-dimethylformamide and tetramethylthiuram disulfide), and this compound can be an indicator of the fungal biomass in polluted soils or used to monitor bioremediation processes.

**Figure 2:**
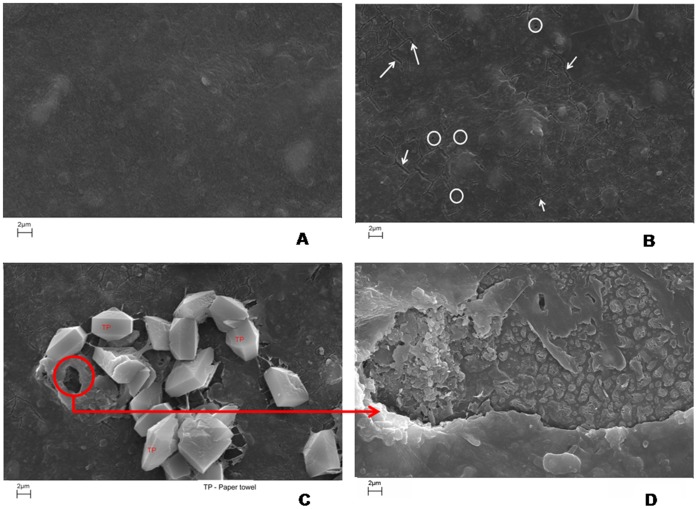
Scanning electron microscopy of oxo-biodegradable plastic strip without fungus (A) and after 45 days of colonization with *Pleurotus ostreatus* PLO6 (B, C and D), showing the cracks (arrows) and holes (circles). TP – Paper towel.

**Figure 3:**
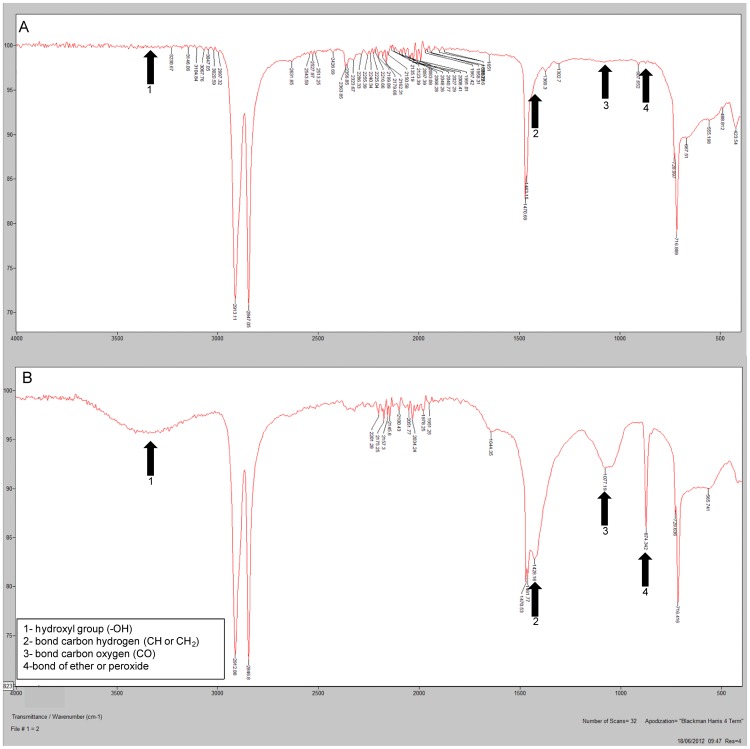
Espectrum of Fourier transform infrared spectroscopy of the oxo-biodegradable plastic strip without fungus (A) and after 45 days of colonization with *Pleurotus ostreatus* PLO6 (B).

**Figure 4:**
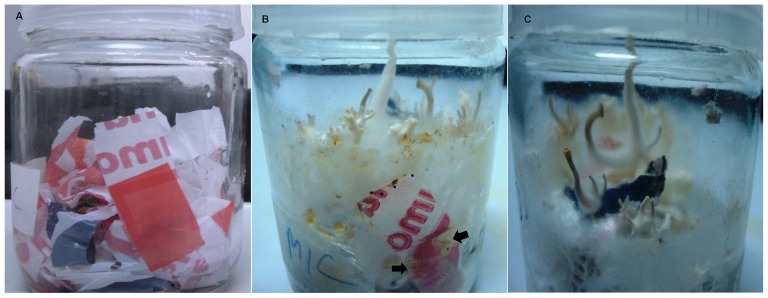
Oxo-biodegradable plastic strip without fungus (A) and after 45 days of colonization with *Pleurotus ostreatus* PLO6, showing halos (B) of discoloration (arrows) and mushrooms (C).

The degradation of oxo-biodegradable waste by *P. ostreatus* was also evidenced by the formation of cracks and holes in the plastic surface after 45 d of incubation (Figure 2) and by the formation of hydroxyl groups (−OH) and carbon-oxygen bonds (Figure 3). Although these changes are not evidence of degradation in metabolic terms, the visual and microscopic changes in the plastic waste, such as wrinkles on the surface, formation of holes and cracks, crumbling (Figure 2), discoloration or changes in color and biofilm formation (Figure 4), can be used as evidence of microbial degradation [15]. These types of alterations were also reported by Bonhomme et al. [12] and Shah [15]. Furthermore, a decrease in the mechanical proprieties of the oxo-biodegradable polymers was observed after fungal growth (Table 1). These results show that the polymers are more fragile, less resistant to the breaks or fragmentations and, when discarded in the environment, can be more susceptible to mineralization than those waste plastics without fungal treatment.

The primary chemical changes that were observed during the degradation of the oxo-biodegradable waste were the oxidation reactions, as evidenced by the formation of hydroxyl groups (Figure 3, band among 3500–3200 cm^−1^), the formation of carbon-oxygen bonds (Figure 3, band in 1077 cm^−1^) and the formation of ether or peroxide groups (Figure 3, band in 974 cm^−1^). Furthermore, we observed the formation of a carbon-hydrogen bond (Figure 3, band in 1428 cm^−1^ of CH or CH_2_). The oxidation of the low-density polyethylene and the high-density polyethylene by microorganisms was also shown by Chiellini et al. [24] and Sudhakar et al. [16]. The formation of the hydroxyl groups was also observed during poly(vinylacetate) degradation by lipase [29] and polyethylene degradation by *R. rhodochrous* and *C. cladosporoides* [12].

During the incubation period, the formation of halos of discoloration in the plastic waste was also observed when compared to the control (Figures 4A and 4B). These halos may be caused by the activity of the lignocellulolytic enzymes secreted by the fungus. Several authors have observed the activity of these enzymes in the decolorization of industrial dyes [29]–[34]. Although not related to the degradation of plastic waste, the observation of the formation of halos of discoloration is important because it is common plastic bags that are used in supermarkets in the presence of different dyes.

The formation of the halos of discoloration (Figure 4A and 4B) and the degradation of plastics (Figure 2, Figure 3) may be due to lignocellulolytic enzyme activity (Table 2). Extracellular microbial enzymes, such as depolymerases and esterases, are involved in the degradation of plastic polymers, as observed by Shah et al. [15] and Gu [35]. These enzymes cleave the polymer chain in compounds or small molecules to form oligomers, monomers and dimers, which can be assimilated by microorganisms [35]. Roldán-Carrillo et al. [36] observed a 74% degradation of plastic polymers that are based on starch by the activities of amylase, cellulase, lignin peroxidase and MnP produced by *Phanerochaete chrysosporium*. However, in our study, we did not observe MnP activity (Table 2), which can be due to (a) the low synthesis of this enzyme or (b) the low availability of enzyme cofactors, such as manganese, in the culture medium. MnP is a glycoprotein that contains iron as the prosthetic group and is dependent on the hydrogen peroxide and manganese concentration for lignin oxidation [37].

The oxidation of the oxo-biodegradable plastic observed in this study (Figure 4) was mainly due to laccase activity (Table 2), in the process known as cometabolism. Sivan [38] also observed that laccase produced by *Rhodococcus ruber* C208 is involved in polyethylene biodegradation and that it may play a role in the oxidation of the hydrocarbon backbone of polyethylene. Laccase activity includes the oxidation of the phenol and non-phenol components of the lignin and the formation of hydroxyl groups and free radicals during the degradation of lignocellulosic compounds and other compounds, such as malathion, polycyclic aromatic hydrocarbons and phthalocyanine [39]–[42]. Cometabolism is a process that is commonly used in the bio-treatment of recalcitrant compounds, where a carbon source is added to induce the synthesis of a certain enzyme and the activity of this enzyme degrades not only the source added but also the other compounds that are present in the substrate, including the pollutant [42], [43]. In our study, the paper towel was used as the carbon source to induce the synthesis of the lignocellulolytic enzymes. Furthermore, the addition of the paper towel in the substrate was important to maintain the moisture and the initial fungal growth because in the treatment without the addition of the paper towel, there was a rapid moisture loss, and no mycelial growth was observed.

The degradation of the oxo-biodegradable polyethylene (Figure 2 and 3) that has little or no similarity to the enzymatic substrate of laccase may have been due to the enzymatic reaction of the degradation of the paper towel or of the dyes found on the surface of the plastic waste (Figure 3 and 4). This reaction may have generated intermediate loading, which caused the reduction/oxidation of the pro-oxidant (Table 1), and this reduction/oxidation led to polyethylene chain breakage. This mechanism suggested can really have happened because the holes in the surface of the plastic and the fragments of paper towel are overlapping (Figure 2C). However, the degradation mechanism of this polymer by *P. ostreatus* may have been more complex and may have included both enzymatic and non-enzymatic reactions. This mechanism can be elucidated using a chemical tag on the purified enzyme and the pro-oxidants.

The higher activity of the cellulase and xylanase compared with the activity of the laccase (Table 2) may be due to the content of the cellulose and hemicellulose found in the paper towel that was added to the growth substrate. The activity of cellulose and xylanase is related to the hydrolysis of the β-glycosidic bond of the cellulose and hemicellulose, respectively [44].

Our results provide the first evidence of *P. ostreatus* growth and mushroom formation by the degradation of a biodegradable plastic in the relatively short period of 45 d (Figures 2, 4B and 4C). This finding reveals new possibilities for the treatment of domestic waste derived from biodegradable plastics by composting with white rot fungi. Moreover, this process represents a new option for the management of plastic waste arising from industrial plastic packaging, food packaging, and plastic supermarket bags.

## Conclusion

*P. ostreatus* is capable of degrading oxo-biodegradable plastic and producing mushrooms using the plastic waste without any prior physical treatment. The results of this study are important for elucidating the biodegradation process of plastic waste and revealing a new alternative for the proper treatment of these pollutants.

## Supporting Information

Figure S1 Espectrum of Fourier transform infrared spectroscopy of the oxo-biodegradable plastic bags (sample) that were used as substrate to growth of *Pleurotus ostreatus* PLO6, compared to the library spectra.
(TIF)

Figure S2 Espectrum of diffraction in ray X on the surface of oxo-biodegradable plastic strip, before of incubation with *Pleurotus ostreatus* PLO6. Red spots (titanium oxide).
(TIF)
