# Supplementary figures and images for: Degradation of Oxo-Biodegradable Plastic by Pleurotus ostreatus
Source: PLoS One. 2013 Aug 15;8(8):e69386. doi: 10.1371/journal.pone.0069386 (PMC3744528; doi:10.1371/journal.pone.0069386)

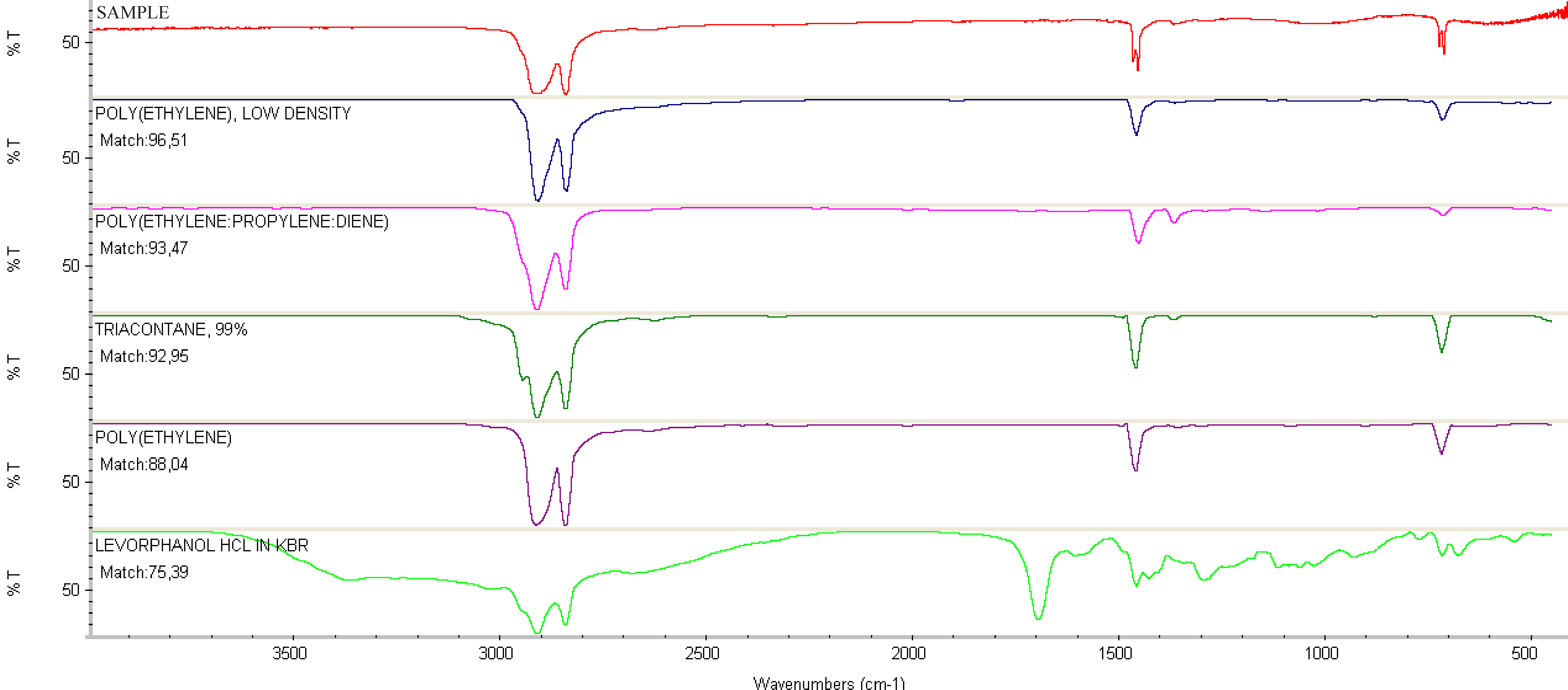

Supplement: Figure S1 — Espectrum of Fourier transform infrared spectroscopy of the oxo-biodegradable plastic bags (sample) that were used as substrate to growth of Pleurotus ostreatus PLO6, compared to the library spectra. (TIF) [file pone.0069386.s001.tif]

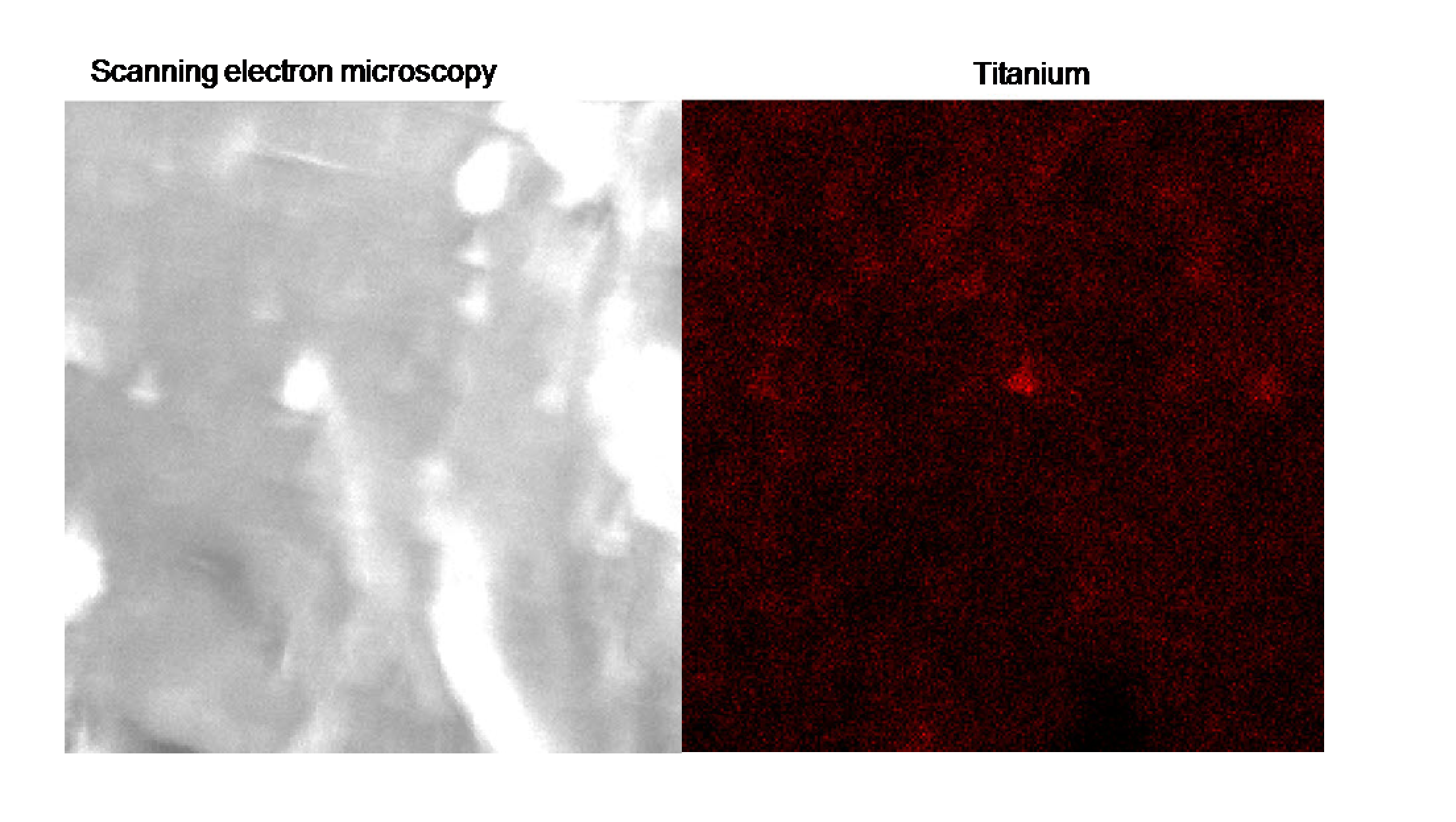

Supplement: Figure S2 — Espectrum of diffraction in ray X on the surface of oxo-biodegradable plastic strip, before of incubation with Pleurotus ostreatus PLO6. Red spots (titanium oxide). (TIF) [file pone.0069386.s002.tif]
